# Automated As-Built Model Generation of Subway Tunnels from Mobile LiDAR Data

**DOI:** 10.3390/s16091486

**Published:** 2016-09-13

**Authors:** Mostafa Arastounia

**Affiliations:** Department of Geomatics Engineering, University of Calgary, 2500 University Drive NW, Calgary, AB T2N 1N4, Canada; marastou@ucalgary.ca; Tel.: +1-403-210-7140

**Keywords:** LiDAR, modeling, tunnel, laser scanning, mobile mapping

## Abstract

This study proposes fully-automated methods for as-built model generation of subway tunnels employing mobile Light Detection and Ranging (LiDAR) data. The employed dataset is acquired by a Velodyne HDL 32E and covers 155 m of a subway tunnel containing six million points. First, the tunnel’s main axis and cross sections are extracted. Next, a preliminary model is created by fitting an ellipse to each extracted cross section. The model is refined by employing residual analysis and Baarda’s data snooping method to eliminate outliers. The final model is then generated by applying least squares adjustment to outlier-free data. The obtained results indicate that the tunnel’s main axis and 1551 cross sections at 0.1 m intervals are successfully extracted. Cross sections have an average semi-major axis of 7.8508 m with a standard deviation of 0.2 mm and semi-minor axis of 7.7509 m with a standard deviation of 0.1 mm. The average normal distance of points from the constructed model (average absolute error) is also 0.012 m. The developed algorithm is applicable to tunnels with any horizontal orientation and degree of curvature since it makes no assumptions, nor does it use any a priori knowledge regarding the tunnel’s curvature and horizontal orientation.

## 1. Introduction

The safety of transportation tunnels (such as subway tunnels) is of great importance. Therefore, they need to be regularly monitored to ensure that they are neither deformed, nor displaced excessively. The tunnels are traditionally monitored by regular measurement of a few benchmarks, employing land surveying instruments such as total stations. The traditional technique in which very few benchmarks are recorded is quite time-consuming and costly. As a result, the redundancy of the acquired data is quite low implying that its reliability is also low. Furthermore, the traditional methods require physical presence of land surveyors in the tunnel, which interrupts the regular train schedule. Poor environmental conditions in tunnels can also negatively affect the quality of the collected data [1].

Mobile mapping technology, on the other hand, does not have any of the abovementioned shortcomings since mobile laser scanning (MLS) systems provide quite fast data collection with very high redundancy, which improves the reliability of the acquired data. Moreover, poor environmental conditions do not have a negative impact on MLS systems since they collect data automatically by using active Light Detection and Ranging (LiDAR) sensors. However, the processing of large-volume MLS data can be time-consuming and expensive. Automation of MLS data processing increases its pace and decreases the associated costs. As a result, deformation and displacement analyses can be carried out more frequently, which enhances the safety of tunnels.

This study aims to demonstrate a workflow to automatically create as-built models of tunnels from MLS data. As-built models can be utilized for three salient applications: construction progress monitoring (for project management); deformation and displacement analysis (maintenance applications); and planning for future design enhancements. The construction progress can be monitored by comparing the as-built model of a tunnel under construction with the associated as-designed model. The deformation and displacement are analyzed by comparing the as-built models of a tunnel from two (or more) different epochs. Finally, an as-built model can be utilized for the planning of future design enhancements since it provides an accurate and precise survey of the current state of a tunnel. The main focus of this work is the generation of such as-built models. The following introduces the typical structure of subway tunnels as the required background knowledge. Section 2 reviews the most relevant studies in this domain, which is followed by description of test dataset used in this study in Section 3. The methodology is explained in Section 4. Section 5 discusses the achieved results and the conclusions are presented in Section 6.

**Figure 1:**
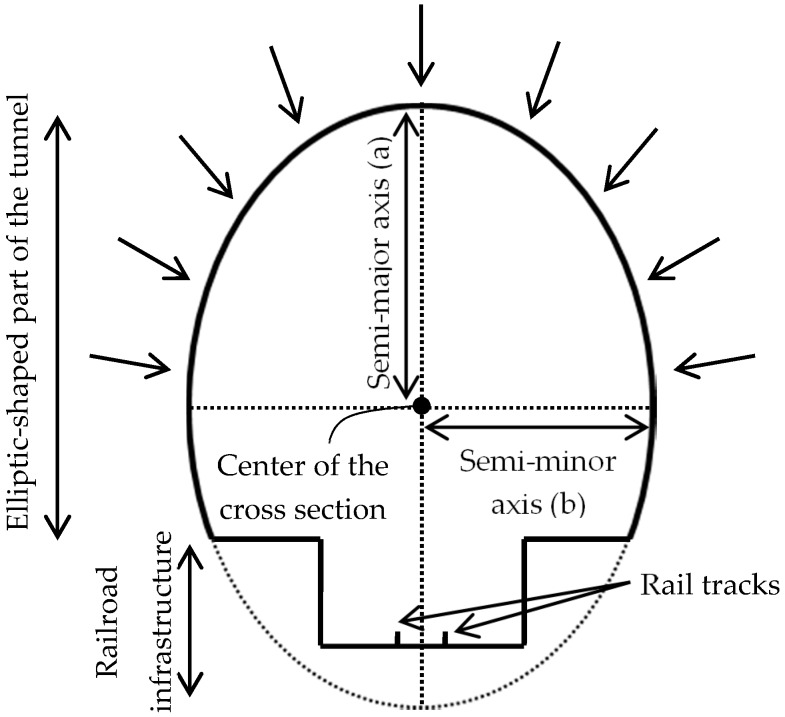
The cross sections of subway tunnels consist of two parts: the upper elliptic-shaped part; and the lower part constituting the railroad infrastructure. The arrows represent the weight of the above terrain, which is loaded on the upper part of the tunnel cross sections.

Subway tunnels have a standard tubular physical shape. As is evident in Figure 1, the cross sections of subway tunnels have two primary parts: the upper part with an elliptic shape and the lower part constituting the railroad infrastructure. Though both bottom and upper parts deform under the terrain weight load, the bottom part will act more stiffly due to the larger cross section of the rail infrastructure. Therefore, the most relevant deformations will take place in the upper (elliptic-shaped) part, since only this area is part of the exterior structural support system of the tunnel, so only the upper elliptic-shaped part of the tunnel is considered for the construction of the as-built model. A crucial part of the developed algorithm is extraction of the tunnel cross sections since deformation and displacement analyses are performed on the basis of the cross sections [1]. Cross sections are orthogonal to the tunnel’s main axis and are identified by their normal vector, semi-major axis, semi-minor axis, and the coordinates of their center. The tunnel main axis is defined as a set of points lying on the center of cross sections. It is required to note that the lower part of Figure 1 displays a sample railroad infrastructure, which may have different configurations in different subway tunnels. However, these differences in configuration are not relevant to the current study.

## 2. Literature Review

MLS systems have two primary units: an imaging unit (LiDAR sensor); and a navigation unit. LiDAR sensors are active sensors that collect data of visible sites and objects by laser range finding. They measure either the time of flight of each emitted laser pulse or the phase shift of the modulating envelope in order to derive the range. They are non-contact measurement instruments that produce a three-dimensional (3D) representation of objects called a point cloud i.e., a set of points with 3D coordinates in a global or national coordinate system. LiDAR sensors can be mounted on terrestrial/airborne and static/mobile platforms. On static terrestrial platforms, the surrounding environment is scanned by rotation of the instrument head around its vertical axis and deflection of the laser beam in a vertical plane using a rotating mirror. On mobile platforms, the LiDAR sensor is attached to a moving vehicle and acquires 3D geometrical information in two-dimensional (2D) profiles along the vehicle’s trajectory. Terrestrial LiDAR data typically provides higher accuracy and point sampling due to its shorter spatial offset from objects of interest, compared to airborne LiDAR data. Many researches have recently studied object extraction and modeling from LiDAR data in various environments. Dorninger and Pfeifer [2], Pu and Vosselman [3], and Kim and Habib [4] extracted and modeled buildings from LiDAR data. Jaakkola et al. [5] and Yan et al. [6] extracted and modeled road and road markings from mobile LiDAR data. Arastounia and Lichti [7] automatically recognized key components of electrical substations from terrestrial LiDAR data. Leslar et al. [8] employed mobile LiDAR to create asset inventory of railroad corridors. Oude Elberink and Khoshelham [9] and Arastounia [10] modeled railroad corridors exploiting mobile LiDAR data.

The following reviews the studies that specifically focus on the extraction and modeling of tunnels from LiDAR data. Tsakiri et al. [11] and Wang et al. [12] discussed the potential of LiDAR technology for deformation analysis of various objects comprising tunnels. They also proposed some surface modeling approaches for deformation analysis. Gosliga et al. [13] analyzed the deformation of a tunnel by directly fitting a circular cylinder to a tunnel point cloud acquired by a terrestrial laser scanning (TLS) system. Their proposed algorithm can only be applied to straight tunnels with no curvature. Moreover, a grid is fitted to the data in this work and the deformation is analyzed at the grid cell level, which leads to less accurate results. Vezočnik et al. [14] inspected the displacement of underground pipelines. LiDAR technology, global navigation satellite system (GNSS), and precise tacheometry are employed to compute the coordinates of some “representative” points on the pipelines. The pipeline displacement is then studied by monitoring the position of the representative points over a period of time. Qiu and Wu [1] employed an automatic electronic total station (ETS) for tunnel deformation analysis. Although using an ETS reduces the data collection time, the redundancy of data is still much less than that of MLS systems. Delaloye et al. [15] compared the traditional techniques (using total stations) with new techniques (employing LiDAR sensors) for deformation analysis of tunnels. They recommended employing LiDAR sensors since they provide fast data acquisition and full surface characterization and they do not need geo-referencing. Goncalves et al. [16] inspected the cross sectional deformation of a subway tunnel from MLS data by employing image processing techniques. Once the cross sections are extracted, they are converted into 2D images, which are further analyzed by image processing techniques. Han et al. [17] studied the deformation of a road tunnel in which the extraction of cross sections is carried out manually. The deformation is also analyzed by inspecting the displacement of some corresponding points from two different scans. They improved this work by eliminating the cross section extraction step in Han et al. [18]. Kang et al. [19] extracted the cross sections of a circular subway tunnel from TLS data. While the cross sections are successfully extracted, their proposed algorithm is limited to tunnels with circular cross sections. They also employed quadric parametric surface modeling, which makes the algorithm computationally intense and impartial for large datasets such as those obtained using MLS systems. Zai et al. [20] analyzed the deformation of a very small part of a road tunnel using TLS data. They fitted a grid to the tunnel and inspected the displacement of the cell centers from two different epochs. Walton et al. [21] employed the geometric genetic algorithm proposed by Ray and Srivastava [22] in tunnel deformation analysis. Nuttens et al. [23,24,25] employed a TLS system to inspect the deformation of a tunnel from immediately after placement until three months after construction; investigate a tunnel’s deformation under the influence of estuarine tides; and monitor the ovalization of a tunnel, respectively.

The above-mentioned studies utilize two primary approaches for deformation analysis: recording benchmarks, or employing as-built models. The former studies the deformation of a very small portion of a tunnel while the latter portrays a thorough and precise picture of the current state of the tunnel so that the deformation of each and every part of the tunnel can be fully investigated. However, the above studies that pursued the modeling approach do not propose a comprehensive solution as they limit their scope to tunnels with a very specific shape and/or horizontal orientation or they process the data manually. This contribution aims to create the as-built model of subway tunnels in a fully-automated way with no assumptions or a priori knowledge regarding the curvature and horizontal orientation of the tunnel. Furthermore, MLS data is employed in this study due to its fast acquisition and very high redundancy. Only geometrical information (i.e., 3D coordinates of points) is utilized for the data processing and the data was processed in its original 3D format to enhance the processing efficiency.

## 3. Dataset

**Figure 2:**
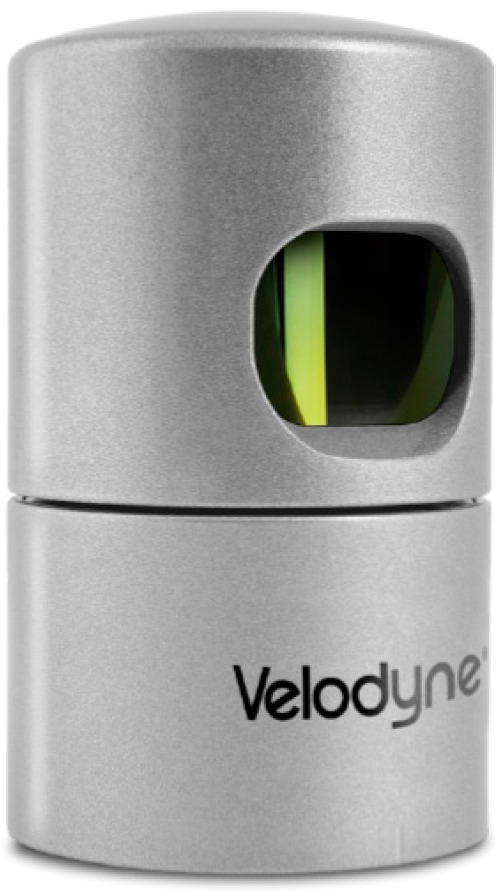
Velodyne 32E LiDAR sensor, employed in this study.

The dataset was collected by employing a Velodyne HDL 32E LiDAR sensor (Figure 2) that was placed on a rail car and scanned the subway tunnel when the train was in motion at 65 km/h. The specifications of the employed sensor are summarized in Table 1. The positional accuracy in Table 1 indicates the accuracy of the measurements made by sensor. Herein, the 0.02 m positional accuracy implies a 68% confidence interval (one standard deviation) on either side of the measured coordinates [26].

**Figure 3:**
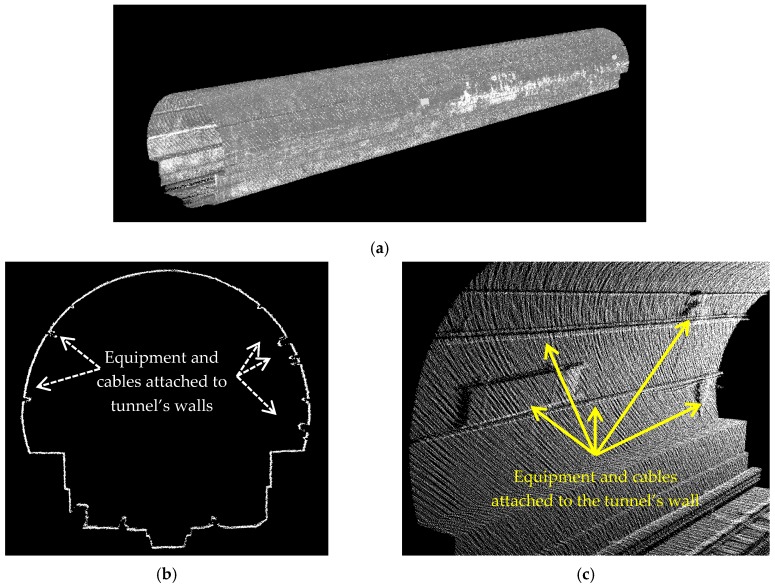
The dataset: (**a**) The entire dataset in an oblique view; (**b**) A side view of the tunnel cross section. The upper part is elliptic-shaped and the lower part constitutes the railroad infrastructure; (**c**) The equipment attached to the tunnel’s wall making cross sectional shape slightly different from a perfect ellipse.

The dataset covers 155 m of a one-way subway tunnel containing more than six million points (6,012,406 points to be precise). The tunnel is along an arbitrary horizontal orientation i.e., it is not along any of cardinal directions. The tunnel has an approximate 7.4° vertical slope resulting in roughly a 20 m height difference between its two ends. The dataset contains geometrical information (3D coordinates of points) and intensity information i.e., a quantized measure of the reflected laser beam power. However, only geometrical information is utilized for the processing in this work and the intensity information is utilized only to provide an appropriate visualization of the dataset. Figure 3a shows the entire dataset in an oblique view; Figure 3b portrays the front view of a cross section; and Figure 3c indicates the equipment and cables attached to the tunnel’s walls that make the cross sectional shape slightly different from a perfect ellipse.

The LiDAR sensor employed to acquire data is an oblique viewing sensor and the point spacing is consequently non-uniform in various parts of the tunnel. Table 2 presents an average point sampling on various parts of the tunnel. As is evident in this table, employing an oblique-viewing LiDAR sensor results in lower point sampling on the ceiling and the floor, compared to the tunnel’s side walls. In addition, no blind spots or occlusions by the support system of the employed LiDAR sensor were observed in the dataset.

## 4. Methodology

**Figure 4:**
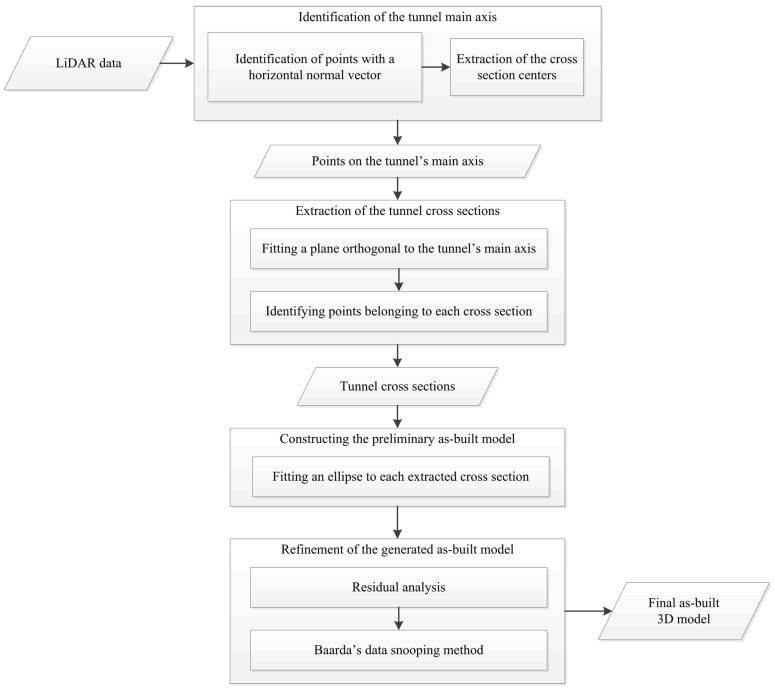
Flowchart of the developed methodology.

The proposed methodology consists of four parts. First, the tunnel’s main axis is automatically identified. Second, the points belonging to each cross section are extracted and next, the as-built model is generated and refined. Finally, the constructed model is evaluated. Figure 4 shows a flowchart for the developed methodology.

### 4.1. Identification of the Tunnel’s Main Axis

The tunnel’s main axis is found by connecting points on the center of the cross sections. First, a 3D plane is fitted to each 3D local spherical neighborhood with a radius of one meter using a parametric Least Squares adjustment [27]. The general equation of a 3D plane is:

(1) $$N_{x}x+N_{y}y+N_{z}z-D=0\Rightarrow N_{x}^{\prime }x+N_{y}^{\prime }y+N_{z}^{\prime }z-1=0\Rightarrow N_{x}^{\prime }x+N_{y}^{\prime }y+N_{z}^{\prime }z=1$$

where: $N_{x}^{\prime }=\frac{N_{x}}{D},\;N_{y}^{\prime }=\frac{N_{y}}{D}\;\mathrm{and}\;N_{z}^{\prime }=\frac{N_{z}}{D}$

$N_{x}$, $N_{y}$, $N_{z}$, represent components of the plane’s normal vector ($\vec{N}$) along the cardinal directions i.e., $\vec{N}=\left(N_{x},N_{y},N_{z}\right)$ and *D* is the normal distance of the plane from the coordinate system center. The general equation of parametric least squares adjustment and its closed-form solution are presented in Equations (2) and (3), respectively [27]:

(2) $$A_{n\times u}X_{u\times 1}=Y_{n\times 1}$$

(3) $$X={\left(A^{T}PA\right)}^{-1}A^{T}PY$$

where *A*, *X* and *Y* are the design, unknowns and constants matrices, respectively. *n* is the number of observations (number of data points, in this case) and *u* is the number of unknowns (number of plane parameters, in this case). *P* denotes the weight matrix of observations, which is calculated by multiplication of the a priori variance factor ($\sigma_{0}^{2}$) and the co-factor matrix of the observations ($Q_{l}$) as follows:

(4) $$P=\sigma_{0}^{2}Q_{l}^{-1}$$

The a priori variance factor is set to one ($\sigma_{0}^{2}=1$) and the elements of the co-factor matrix ($\sigma_{P_{i}}$) are equal to the positional accuracy of the employed sensor, which is provided by the sensor manufacturer. Herein, the positional accuracy of the utilized LiDAR sensor is 0.02 m ($\sigma_{P_{i}}=0.02\;m$) that implies a 68% confidence interval (one standard deviation) on either side of the measured coordinates [26], so the co-factor matrix of the observations and weight matrix are as follows:

(5) $$Q_{l}={\left(\begin{array}{ccccc} \sigma_{P_{1}}^{2} & 0 & 0 & 0 & 0\\ 0 & \sigma_{P_{2}}^{2} & 0 & 0 & 0\\ 0 & 0 & . & 0 & 0\\ 0 & 0 & 0 & . & 0\\ 0 & 0 & 0 & 0 & \sigma_{Pn}^{2} \end{array}\right)}_{n\times n}\;\Rightarrow \;P=\sigma_{0}^{2}Q_{l}^{-1}={\left(\begin{array}{ccccc} \frac{\sigma_{0}^{2}}{\sigma_{P_{1}}^{2}} & 0 & 0 & 0 & 0\\ 0 & \frac{\sigma_{0}^{2}}{\sigma_{P_{2}}^{2}} & 0 & 0 & 0\\ 0 & 0 & . & 0 & 0\\ 0 & 0 & 0 & . & 0\\ 0 & 0 & 0 & 0 & \frac{\sigma_{0}^{2}}{\sigma_{Pn}^{2}} \end{array}\right)}_{n\times n}$$

Considering the equation of a 3D plane in Equation (1), the design, unknowns, and constants matrices are:

(6) $$A={\left(\begin{array}{ccc} x_{1} & y_{1} & z_{1}\\ x_{2} & y_{2} & z_{2}\\ . & . & .\\ . & . & .\\ x_{n} & y_{n} & z_{n} \end{array}\right)}_{n\times 3}\;,\;X={\left(\begin{array}{c} N_{x}^{\prime }\\ N_{y}^{\prime }\\ N_{z}^{\prime } \end{array}\right)}_{3\times 1}\;\mathrm{and}\;Y={\left(\begin{array}{c} 1\\ 1\\ .\\ .\\ 1 \end{array}\right)}_{n\times 1}$$

**Figure 5:**
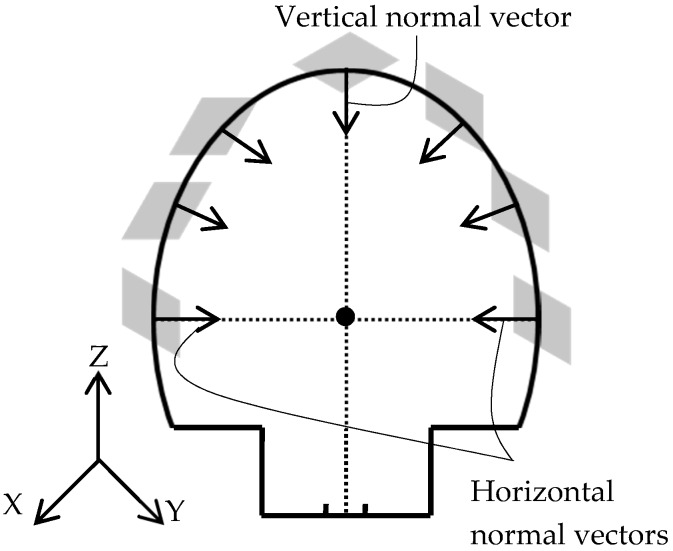
The normal vector of the local best-fit planes on various parts of the tunnel. The grey planes represent the local best-fit planes and the arrows show their normal vectors. Only horizontal and vertical normal vectors go through the cross section’s center (black point).

Thus, the normal vectors of all local spherical neighborhoods are calculated by employing the least squares adjustment (Equation (3)) and matrices in Equations (5) and (6). Only horizontal and vertical normal vectors go through the center of cross sections. This is evident in Figure 5 that indicates a sample cross section with an arbitrary horizontal orientation. The Z component of perfectly horizontal and vertical unit vectors is zero and one, respectively. However, due to measurement inaccuracies, herein, a normal vector is considered horizontal if it satisfies the condition in Equation (7) and is considered vertical if it meets the condition in Equation (8) in which $V_{z}$ represents the Z component of the unit normal vector i.e., $\vec{V}=\left(V_{x},V_{y},V_{z}\right)$. Moreover, if multiple normal vectors are found to meet the conditions in Equations (7) and (8), only the normal vector with the smallest/largest *V_Z_* is considered horizontal/vertical, respectively:

(7) $$|V_{z}|\leq 0.01$$

(8) $$|V_{z}|\geq 0.99$$

**Figure 6:**
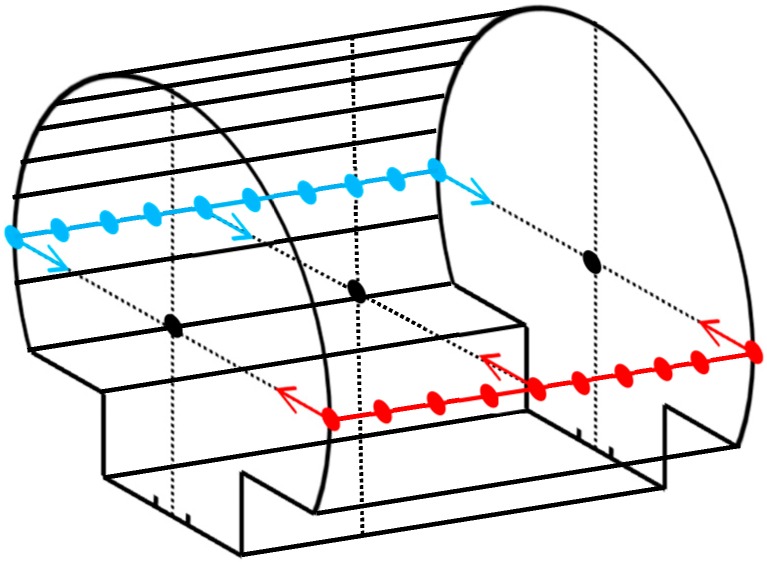
Identification of points on the tunnel’s main axis (black points) by finding the mid-point of two closest points from opposite walls of the tunnel (blue and red points). The arrows represent the normal vector of the local best-fit planes.

That being said, points with horizontal normal vectors that are within one meter 3D Euclidean distance of one another are clustered. This results in two segments (one segment on either side of the tunnel) and each segment contains points with a horizontal normal vector. These two segments are indicated in Figure 6 as one segment in blue and another segment in red on opposite walls of the tunnel (let us call them “blue and red segments”). One should note that Figure 6 illustrates a sample tunnel and the proposed methodology is applicable to tunnels with any degree of curvature since no assumption is made regarding the tunnel’s curvature in the developed algorithm. The one-meter threshold utilized for the neighborhood size of plane fitting and clustering in this section is selected based on the data point sampling, tunnel’s dimensions, and specifications of the employed LiDAR sensor. The neighborhood needs to be large enough to provide reliable information regarding the object of interest. It is also required to include only points belonging to the object of interest and exclude points belonging to other objects. Herein, the employed dataset contains more than five hundred points per square meter (Table 2) implying that a spherical neighborhood with a radius of one meter provides reliable information regarding the normal vector of the local best-fit plane. Furthermore, there are no other external objects in one-meter neighborhood of the tunnel’s walls suggesting that the considered spherical neighborhoods (with a radius of one meter) only include points belonging to the tunnel’s walls. One should note that the presence of points belonging to equipment on the tunnel’s walls (Figure 3b,c) in the considered neighborhoods is inevitable regardless of the neighborhood size since such equipment are attached to the walls. These points will be identified and excluded by further analysis in the following steps (Section 4.3.2) though.

**Figure 7:**
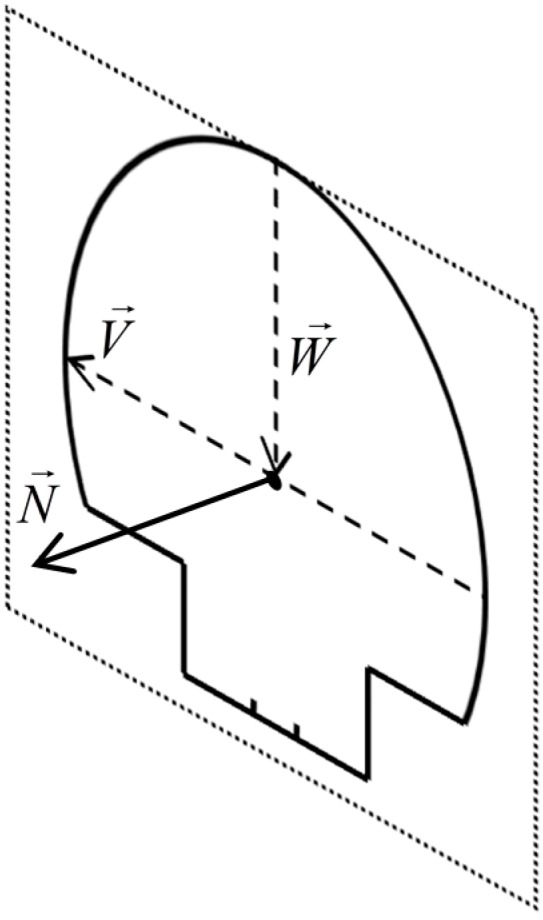
The calculation of a sample cross section’s normal vector (Section 4.1). The dashed rectangle represents the plane fitted to the cross section in Section 4.2.

Next, a point from the blue segment is randomly chosen and its closest point from the red segment on the opposite wall is found. Enforcing the minimum distance between two points from opposite walls ensures that they lie on the “exact” opposite sides of the tunnel implying that their mid-point is located on the center of the cross section, so the center of the query cross section is obtained by calculating the midpoint of two points from the “exact” opposite sides of the tunnel. This process is pursued for all points of the blue and red segments and consequently the center of all cross sections are identified. The tunnel’s main axis is then extracted by connecting the identified centers of the cross sections. Figure 6 shows three sample points on the tunnel’s main axis (black points). Afterwards, the normal vector of each cross section is computed. To this end, the vector connecting two closest points from opposite sides of the tunnel ($\vec{V}$) and the vector connecting the center of cross section to the topmost part of the cross section ($\vec{W}$) are calculated. The topmost point is found by seeking for the point with a vertical normal vector (satisfying the condition in Equation (8)) with the shortest spatial offset from the cross section center. As indicated in Figure 7, vectors $\vec{V}$ and $\vec{W}$ lie on the plane that goes through the cross section so their cross product defines the cross section’s normal vector ($\vec{N}$):

(9) $$\vec{N}=\vec{V}\times \vec{W}$$

### 4.2. Extraction of the Tunnel Cross Sections

The cross sections are extracted by fitting 3D planes orthogonal to the tunnel’s main axis and identifying points within a certain distance from the fitted planes. The plane fitting is carried out using 3D coordinates of the center (*x*_0_, *y*_0_, and *z*_0_) and normal vector ($\vec{N}$) of the cross sections that were calculated in the previous section. Afterwards, points within 0.05 m normal distance from (either side of) the fitted plane are considered as belonging to the cross section under study. As a result, the tunnel’s cross sections at 0.1 m intervals are extracted. This threshold (0.05 m) is equal to half of the interval of cross section extraction since points within 0.05 m “on either side” of the fitted plane are considered as belonging to the cross section under inspection. That being said, since modeling is carried out at the cross section level, the shortest possible interval of cross section extraction corresponds to the most precise modeling results. The shortest possible interval of cross section extraction (that is twice as large as the query threshold) needs to be selected with respect to the positional accuracy of the employed sensor. Herein, the positional accuracy of the employed sensor (*σ* = 0.02 m) implies a 95% confidence interval of 2*σ* (0.04 m) on either side of the measured coordinates. Thus, the 0.05-m threshold is selected to be slightly larger than 2*σ* (0.04 m). The formula utilized to calculate the normal distance (*D_i_*) between a query point and the fitted plane in indicated in Equation (10) in which *x*_0_, *y*_0_, and *z*_0_ denote the 3D coordinates of the cross section center and *x_i_*, *y_i_*, and *z_i_* denote the 3D coordinates of a query point. In Equation (10), $N_{x},N_{y}\;\mathrm{and}\;N_{z}$, represent the components of the cross section’s unit normal vector:

(10) $$D_{i}=N_{x}(x_{i}-x_{0})+N_{y}(y_{i}-y_{0})+N_{z}(z_{i}-z_{0})$$

### 4.3. As-Built Model Generation

#### 4.3.1. Modeling by Least Squares Adjustment

The ellipse and circle are two common cross sectional shapes of subway tunnels. Considering that a circle is a specific form of an ellipse (with equal semi-major and semi-minor axes), an ellipse is fitted to the points belonging to each extracted cross section using least squares adjustment. Least squares fitting algorithms generally fall into two primary categories: algebraic methods and geometric methods. Algebraic methods are more common due to their simplicity, linear nature, and high computational efficiency. Geometric methods provide a more robust fitting approach by solving a non-linear problem. Though geometric approaches are computationally more expensive, they provide a more reliable solution in the presence of outliers in data [22]. This is important since Least Squares adjustment is quite sensitive to outliers implying that if outliers are not removed, they will have a significant negative impact on the generated model. In subway tunnel point clouds, outliers are introduced by the ever-present equipment attached to the tunnel’s walls (Figure 3b) and random irregularities in the tunnels’ cross sectional shape (Figure 3c), which make the tunnels’ cross sectional shape slightly different from a perfect ellipse. Such outliers are easily identifiable due to their large deviation from the actual data points. Therefore, herein, an algebraic (linear) least squares fitting method is employed, which is followed by sequential application of residual analysis and Baarda’s data snooping method [28] to refine the modeling parameters (Section 4.3.2). This results in a precise modeling as well as a computationally efficient algorithm since a linear least squares adjustment is applied to the outlier-free data.

Fitting an ellipse to a cross section with an arbitrary orientation requires the general form of quadric surfaces (Equation (11)) and application of complicated constraints [29,30,31,32], which makes the algorithm computationally very intense. However, if the normal vector of the cross section is along one of the cardinal directions, a simple ellipse model can be employed rather than the general form of quadric surfaces. Employing an ellipse model significantly improves the computational efficiency since the number of unknowns decreases from ten parameters (coefficients of Equation (11)) to two parameters (semi-major and semi-minor axes of the ellipse). Thus, each cross section is automatically rotated so that its normal vector ($\vec{N}$) is aligned along a cardinal direction (in this case Y axis) in the dataset’s coordinate system. The rotation is carried out by two sequential rotations: one rotation around Z axis; and one rotation around X axis, as in Equation (12). The rotation is automated since the rotation angles (*θ_z_* and *θ_x_*) are computed using the normal vector ($\vec{N}$) of cross sections, which were calculated in the previous section. The rotation is executed as in Equation (12) in which *R_x_* and *R_z_* denote the rotation matrices around X and Z axis, respectively. *P_i_* denotes 3D coordinates of a sample point and $P_{i}^{\prime }$ represents the respective rotated coordinates:

(11) $$Ax^{2}+By^{2}+Cz^{2}+Dxy+Exz+Fyz+Gx+Hy+Iz+J=0$$

(12) $$\underset{P_{i}^{\prime }}{\underset{︸}{\left(\begin{array}{c} x_{i}^{\prime }\\ y_{i}^{\prime }\\ z_{i}^{\prime } \end{array}\right)}}=\underset{R_{x}}{\underset{︸}{\left(\begin{array}{ccc} 1 & 0 & 0\\ 0 & \cos(\theta_{x}) & \cos(\theta_{x})\\ 0 & \cos(\theta_{x}) & \cos(\theta_{x}) \end{array}\right)}}\underset{R_{z}}{\underset{︸}{\left(\begin{array}{ccc} \cos(\theta_{z}) & \cos(\theta_{z}) & 0\\ \cos(\theta_{z}) & \cos(\theta_{z}) & 0\\ 0 & 0 & 1 \end{array}\right)}}\;\underset{P_{i}}{\underset{︸}{\left(\begin{array}{c} x_{i}\\ y_{i}\\ z_{i} \end{array}\right)}}$$

where:

(13) $$\theta_{z}=\arctan(\frac{N_{x}}{N_{y}})$$

(14) $$\theta_{x}=\arctan(\frac{N_{z}}{\sqrt{N_{x}^{2}+N_{y}^{2}}})$$

Afterwards, an ellipse is fitted to the upper part of each and every cross section using the least squares adjustment. The upper part is defined as points (belonging to the queried cross section) whose height is equal to or higher than height of the cross section center. The equation of an ellipse orthogonal to Y axis is shown in Equation (15) and the associated design, unknowns, and constants matrices are presented in Equation (16) in which *a* denotes the semi-major axis of the fitted ellipse; *b* represents the semi-minor axis of the fitted ellipse; $x_{i}$ and $z_{i}$ are the coordinates of data points; and *n* is the total number of data points belonging to the queried cross section:

(15) $$\frac{z^{2}}{a^{2}}+\frac{x^{2}}{b^{2}}=1\overset{c=\frac{1}{a^{2}}\;\mathrm{and}\;d=\frac{1}{b^{2}}}{\to }cz^{2}+dx^{2}=1$$

(16) $$A={\left(\begin{array}{cc} z_{1}^{2} & x_{1}^{2}\\ z_{2}^{2} & x_{2}^{2}\\ . & .\\ . & .\\ z_{n}^{2} & x_{n}^{2} \end{array}\right)}_{n\times 2}\;,\;X={\left(\begin{array}{c} c\\ d \end{array}\right)}_{2\times 1}\;\mathrm{and}\;Y={\left(\begin{array}{c} 1\\ 1\\ .\\ .\\ 1 \end{array}\right)}_{n\times 1}$$

The semi-major axis (a) and semi-minor axis (b) of all cross sections are then calculated by applying Least Squares adjustment (Equation (3)) to the matrices in Equation (16). Next, the preliminary model is generated by calculating the coordinates of its points using Equation (17) and the calculated parameters i.e., 3D coordinates of the cross section center (*x*_0_, *y*_0_ and *z*_0_) and the associated axes (*a* and *b*).

(17) $$\frac{{\left(z-z_{0}\right)}^{2}}{a^{2}}+\frac{{\left(x-x_{0}\right)}^{2}}{b^{2}}=1\to \{\begin{array}{cc} z_{i}=z_{0}+a\cos(\theta_{i}) & 1^{\circ }\leq \theta_{i}\leq 360^{\circ }\\ x_{i}=x_{0}+b\sin(\theta_{i}) & 1^{\circ }\leq \theta_{i}\leq 360^{\circ }\\ y_{i}=y_{0} & \end{array}$$

It is required to note that the rotation angles of each cross section are specifically computed using the components of the respective normal vector (Equations (13) and (14)). This implies that the modeling is carried out at the cross section level and makes no assumption regarding the tunnel’s curvature and that is why the developed algorithm is applicable to tunnels with any degree of curvature.

#### 4.3.2. Model Refinement

Once the preliminary model is constructed, the residuals are computed using Equation (18) and the 90th percentile residual of each cross section is calculated as the cut-off threshold. Then, points whose residual is larger than this threshold are identified as outliers and are removed:

(18) V=Y−AX

Afterwards, Baarda’s data snooping method is used to remove the remaining outliers based on standardized residuals, which are computed as follows:

(19) $$nV_{i}=\frac{\left|V_{i}\right|}{\sigma_{0}\times \sqrt{Q_{V}(i,i)}}$$

where *V_i_* and *nV_i_* are the residual and standardized residual of ith point and *Q_V_*(*i,i*) represents the element on the ith row and ith column of the co-factor matrix of residuals (*Q_V_*). The co-factor matrix of residuals (*Q_V_*) is calculated in the following equation:

(20) $$Q_{V}=Q_{l}-A{\left(A^{T}PA\right)}^{-1}A^{T}$$

According to Baarda’s method, points whose standardized residual is larger than four are considered as outliers and are not considered for further processing. Therefore, outliers including points belonging to the tunnel’s equipment are identified and eliminated by residual analysis and Baarda’s method. Finally, the least squares adjustment is applied to the outlier-free data and the final as-built model is generated.

Once the modeling is accomplished, the extracted cross sections and their associated models are rotated back to their original orientation so that the original shape, curvature, and orientation of the tunnel are preserved in the constructed as-built model. Moreover, the dataset’s coordinate system does not make any difference in this particular section since this section only calculates the dimensions of each cross section i.e. its semi-major and semi-minor axes. This is carried out by: aligning each cross section along a cardinal direction (Y axis); calculating its dimensions (by ellipse fitting and model refinement); and rotating it back to its original orientation. The rotation of cross sections is applied only to enhance the computational efficiency since it reduces the number of modeling unknowns (coefficients) from ten to two unknowns. Since a circle is a specific form of an ellipse (with equal semi-major and semi-minor axes), the developed algorithm is applicable to transportation tunnels with either elliptic or circular cross sections.

### 4.4. Evaluation of the Generated Model

#### 4.4.1. Dimensions of the Extracted Cross Sections

The dimensions of cross sections can be fully investigated by calculating their area and eccentricity, which indicates the deviation of the tunnel’s cross sectional shape from being circular. Thus, once the semi-major and semi-minor axes and their standard deviations are obtained, the area (*A*) and eccentricity (*e*) of cross sections along with their standard deviations (*σ_A_* and *σ_e_*) are calculated using Equations (21)–(24). The standard deviations of axes are calculated by Equations (25)–(27):

(21) $$A=\frac{\pi ab}{2}$$

(22) $$e=\sqrt{1-\frac{b^{2}}{a^{2}}}$$

(23) $$\begin{array}{c} A=f(a,b)=\frac{\pi ab}{2}\overset{\mathrm{Law}\mathrm{of}\mathrm{Error}\mathrm{Propagation}}{\to }\\ \sigma_{A}^{2}={\left(\frac{\partial f}{\partial a}\right)}^{2}\sigma_{a}^{2}+{\left(\frac{\partial f}{\partial b}\right)}^{2}\sigma_{b}^{2}\to \sigma_{A}=\frac{\pi }{2}\sqrt{\left(b^{2}\sigma_{a}^{2})+(a^{2}\sigma_{b}^{2}\right)} \end{array}$$

(24) $$\begin{array}{c} e=g(a,b)=\sqrt{1-\frac{b^{2}}{a^{2}}}\overset{\mathrm{Law}\mathrm{of}\mathrm{Error}\mathrm{Propagation}}{\to }\sigma_{e}^{2}={\left(\frac{\partial g}{\partial a}\right)}^{2}\sigma_{a}^{2}+{\left(\frac{\partial g}{\partial b}\right)}^{2}\sigma_{b}^{2}\to \\ \sigma_{e}=\sqrt{{\left(\frac{b^{2}}{a^{3}e}\sigma_{a}\right)}^{2}+{\left(\frac{b}{a^{2}e}\sigma_{b}\right)}^{2}}=\frac{b}{a^{2}e}\sqrt{(\frac{b^{2}}{a^{2}}\sigma_{a}^{2})+\sigma_{b}^{2}} \end{array}$$

#### 4.4.2. Precision of the Estimated Parameters

Once modeling was accomplished, the standard deviation of the semi-major and semi-minor axes is calculated using the following formulas. The standard deviation of the axes are quite important since they indicate how precisely the axes are computed:

(25) $$\left(\begin{array}{cc} \sigma_{c}^{2} & \sigma_{cd}\\ \sigma_{dc} & \sigma_{d}^{2} \end{array}\right)={\overset{⌢}{\sigma }}_{0}^{2}{\left(A^{T}PA\right)}^{-1}$$

(26) $$c=\frac{1}{a^{2}}\to a=f(c)=\frac{1}{\sqrt{c}}\overset{\mathrm{Law}\mathrm{of}\mathrm{Error}\mathrm{Propagation}}{\to }\sigma_{a}^{2}={\left(\frac{df}{dc}\right)}^{2}\sigma_{c}^{2}\to \sigma_{a}=\sqrt{\frac{\sigma_{c}^{2}}{4c^{3}}}$$

(27) $$d=\frac{1}{b^{2}}\to b=g(d)=\frac{1}{\sqrt{d}}\overset{\mathrm{Law}\mathrm{of}\mathrm{Error}\mathrm{Propagation}}{\to }\sigma_{b}^{2}={\left(\frac{dg}{dd}\right)}^{2}\sigma_{d}^{2}\to \sigma_{b}=\sqrt{\frac{\sigma_{d}^{2}}{4d^{3}}}$$

where:

(28) $${\overset{⌢}{\sigma }}_{0}^{2}=\frac{V^{T}PV}{\mathrm{DF}}$$

In the above equations, DF represents the degree of freedom and ${\hat{\sigma }}_{0}^{2}$ denotes the a posteriori variance factor. DF is calculated as the subtraction of unknowns from the number of equations. The number of unknowns of all cross sections is two (semi-major and semi-minor axes) and the number of equations is the number of data points belonging to each cross section. Thus, DF of each cross section is different from others and is computed as the number of data points belonging to each cross section minus 2. The average number of cross section points is 2090 (for the dataset employed), which corresponds to an average DF of 2088. Furthermore, the normal distance of points from the model (i.e., absolute error) is computed as another measure to evaluate the constructed as-built model. The normal distance of points from the model is calculated as in Equation (29) in which *x_i_*, *y_i_* and *z_i_* denote the coordinates of a query point and *x_m_*, *y_m_* and *z_m_* represent the coordinates of the closest point on the model from the query point:

(29) $$Dist=\sqrt{{\left(x_{i}-x_{m}\right)}^{2}+{\left(y_{i}-y_{m}\right)}^{2}+{\left(z_{i}-z_{m}\right)}^{2}}$$

## 5. Results and Discussion

### 5.1. Results of Cross Section Extraction

**Figure 8:**
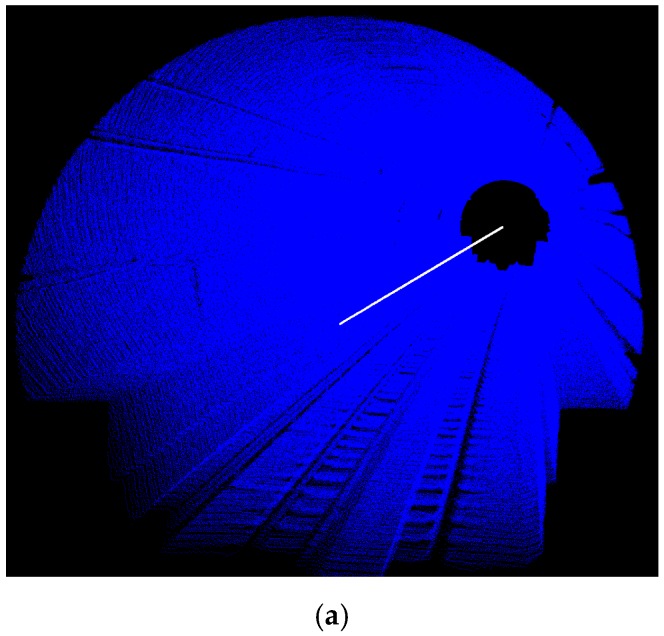

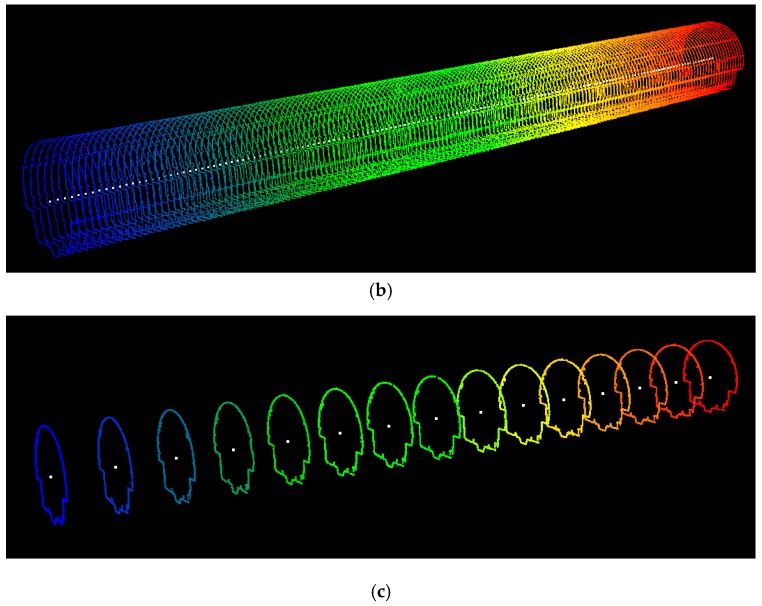
The extracted centerline and cross sections: (**a**) The tunnel’s centerline (center of cross sections) is indicated in white color; (**b**) The extracted cross sections at one-meter intervals; (**c**) The extracted cross sections at ten-meter intervals.

1551 cross sections at 0.1 m intervals were successfully extracted from the dataset described in Section 3. Figure 8 shows the tunnel’s centerline and extracted cross sections at one-meter and ten-meter intervals. As was mentioned in Section 1, the cross section centers (shown as white points) constitute the tunnel’s main axis. It should be noted that the extraction and modeling of cross sections are performed at 0.1 m intervals. However, cross sections at such short interval (0.1 m) are so dense that they cannot be conveniently visualized and differentiated from one another. Thus, Figure 8 depicts the extracted cross sections at larger intervals so that they can be conveniently seen.

**Figure 9:**
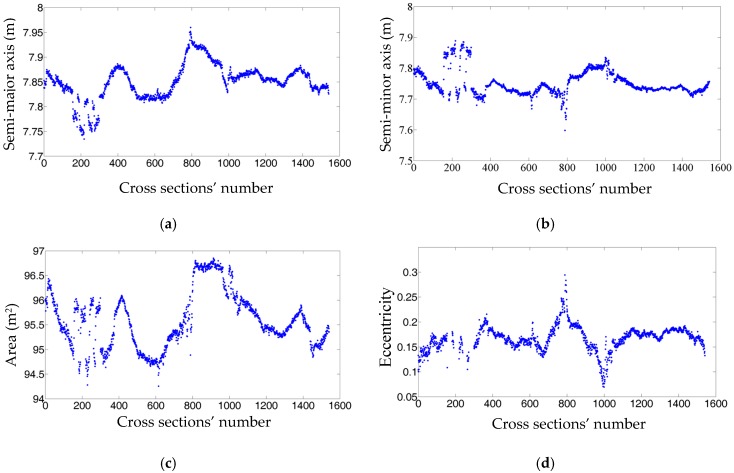
The dimensions of the extracted cross sections: (**a**) Semi-major axis; (**b**) Semi-minor axis; (**c**) Area; (**d**) Eccentricity of the extracted cross sections.

**Figure 10:**
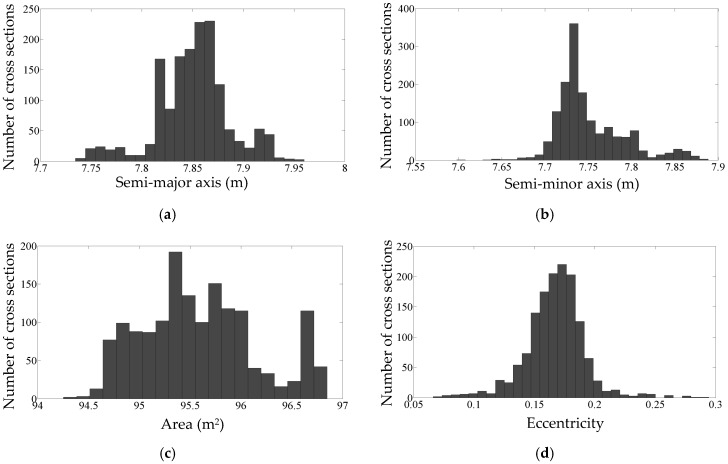
Histogram of the cross sections’ dimensions: (**a**) The histogram of semi-major axis; (**b**) The histogram of semi-minor axis; (**c**) The histogram of area; (**d**) The histogram of eccentricity. The histogram bin width of the axes, area, and eccentricity is 0.01 m, 0.1 m, and 0.01 (unit-less), respectively. The bin widths are selected with respect to the dataset point sampling, the tunnel’s dimensions and the employed LiDAR sensor specifications.

The spatial variations and histograms of the semi-major axis, semi-minor axis, area, and eccentricity of 1551 extracted cross sections are demonstrated in Figure 9 and Figure 10, respectively. The achieved results show that cross sections have an average semi-major axis of 7.8508 m with a standard deviation of 0.2 mm and an average semi-minor axis of 7.7509 m with a standard deviation of 0.1 mm. Their average area and eccentricity are also 95.5838 m^2^ with a standard deviation of 0.0003 m^2^ and 0.17606 with a standard deviation of 0.00002, respectively. Such parameters of a subway tunnel should ideally be constant for all cross sections meaning that the dimensions of cross sections do not vary throughout the tunnel. Although there are some fluctuations in Figure 9, the vast majority of the presented measures have a small deviation from their mean value. This is also evident in Figure 10 that depicts a great portion of semi-major axes are between 7.8 m and 7.9 m and the semi-minor axes predominantly range from 7.7 m to 7.8 m. This indicates that the dimensions of cross sections are quite consistent, considering the positional accuracy of the employed LiDAR sensor (0.02 m). The uniform dimensions of cross sections is confirmed by Figure 9c,d and Figure 10c,d where the area and eccentricity of a large number of cross sections are centered around 95.6 m^2^ and 0.15 (unit-less), respectively. However, a few cross sections with inconsistent dimensions exist, which can be due to one or both of the following two reasons. First, the tunnel’s as-built and as-designed plans might not be fully-compatible suggesting that the tunnel is not constructed exactly according to the designed plans. Second, the tunnel may be experiencing deformation. The former can be investigated by comparing the as-built models with as-designed plans and the latter can be inspected by comparing as-built models of a tunnel from two different epochs. However, neither of them fits into the scope of this study since this work is primarily aimed at automated construction of such as-built models.

Table 3 summarizes the average dimensions of cross sections including the mean and standard deviation of axes, area, and eccentricity. The magnitude of the semi-major and semi-minor axes of each cross section are quite close to one another, implying an elliptic cross sectional shape that is close to a circular shape. This is confirmed by the average eccentricity that is close to zero (0.17606). The information regarding the dimensions of a tunnel can be employed in many applications such as displacement and deformation analysis. Moreover, the calculation of standard deviation of both axes is of great importance when deformation occurs only along one of the axes since such deformations cannot be reflected by the eccentricity.

### 5.2. The Generated As-Built Model

**Figure 11:**
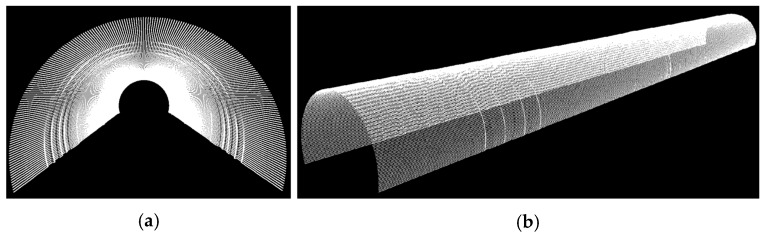
Two different views of the constructed model: (**a**) A front view; (**b**) an oblique view of the model.

Figure 11 demonstrates the front and oblique views of the generated model at 0.1 m intervals.

**Figure 12:**
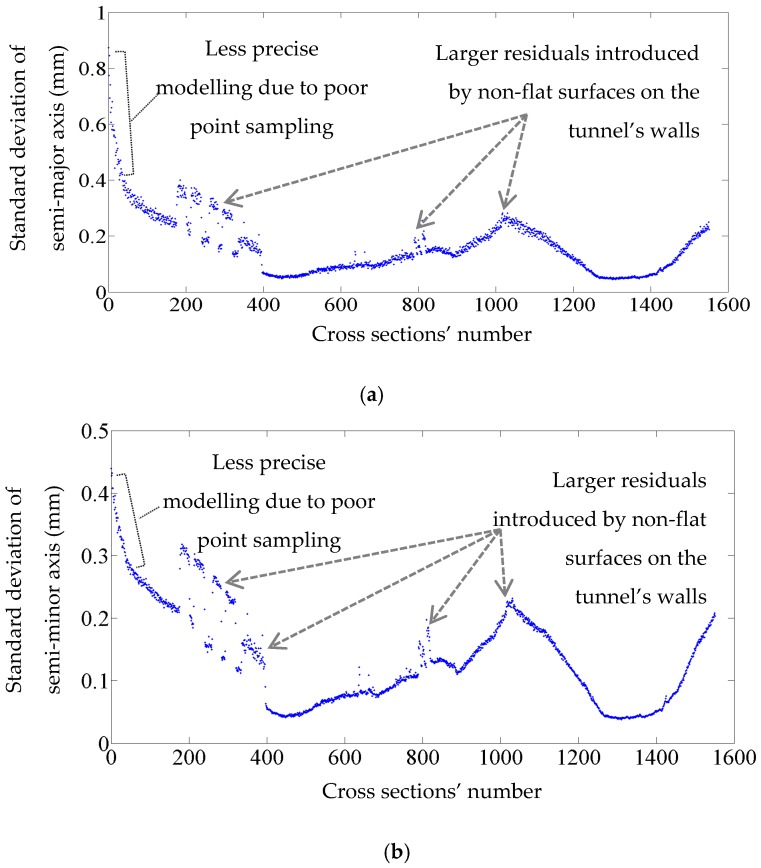
The standard deviation of the cross sections’ axes: (**a**) The standard deviations of semi-major axis; (**b**) The standard deviations of semi-minor axis.

**Figure 13:**
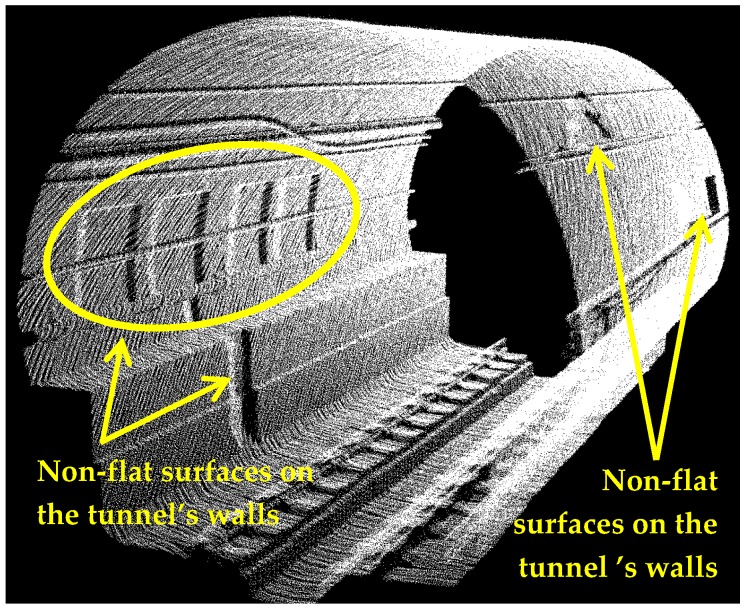
Non-flat surfaces on the tunnel’s walls.

**Figure 14:**
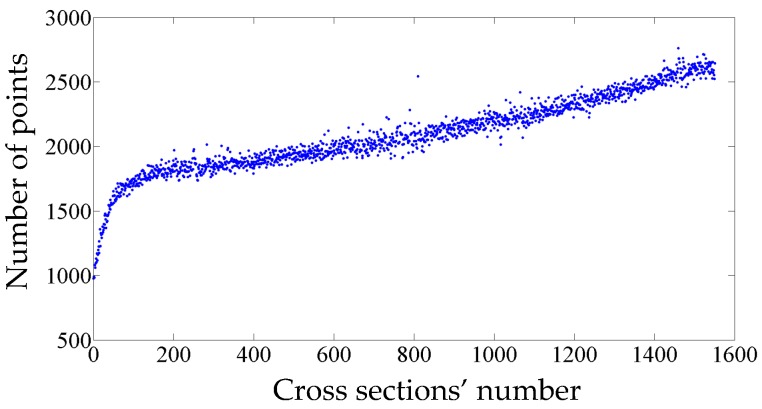
The point sampling of cross sections. The sampling of the first ten meters (corresponding to the first hundred cross sections) is lower than that of the other cross sections.

The standard deviation of the semi-major and semi-minor axes indicates how precisely they are estimated. For instance, the axes of 700th cross section are estimated as 7.8485 m and 7.7364 m with a standard deviation of 0.1 mm for both axes. The standard deviations of all computed axes are indicated in Figure 12. The average standard deviation of semi-major axes and semi-minor axes are 0.2 mm and 0.1 mm, respectively. As is evident in Figure 12, the majority of the standard deviations are quite small (smaller than 0.5 mm) suggesting that the generated model is quite precise. However, some cross sections (indicated with arrows in Figure 12) have slightly higher standard deviations. Such inconsistencies are introduced by non-flat surfaces on some parts of the tunnel, which are displayed in Figure 13. Moreover, the point sampling of the first ten meters of the tunnel is less than the tunnel’s average sampling and this lead to higher standard deviations for the first hundred cross sections (illustrated by dashed grey brackets in Figure 12). The lower sampling of the first ten meters of the tunnel can be visualized in Figure 14 that depicts the point sampling of all cross sections. Furthermore, a gradual increase in the number of points along the length of the tunnel can be observed in Figure 14. The author believes this can be the result of one or both of the following reasons:

1. Speed reduction of the train carrying the LiDAR sensor
2. Change in the setting of the employed LiDAR sensor during data collection

However, the author was not able to verify these assumptions since he was not involved in data acquisition.

**Figure 15:**
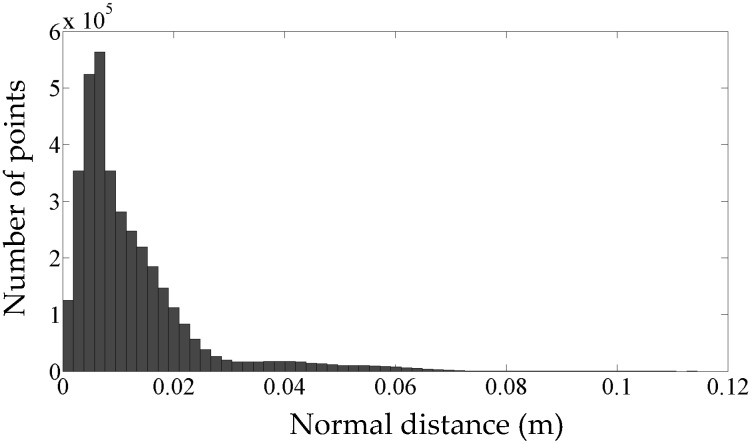
The histogram of normal distance of all points from the constructed model.

Figure 15 shows the histogram of the normal distance of points from the as-built model (absolute errors). Recall that the positional accuracy of the employed LiDAR sensor (*σ* = 0.02 m) implies a 95% confidence interval of 2*σ* (0.04 m) on either side of the measured coordinates. This is confirmed by Figure 15 that indicates 95% of points lie within 0.04 m of the generated model. The average normal distance of all points from the model (i.e., average absolute error) is 0.012 m.

## 6. Conclusions

This contribution presents a novel fully-automated methodology for as-built model generation of subway tunnels employing 3D MLS data. The dataset used in this work covers 155 m of a one-way subway tunnel containing more than six million points, which are acquired by a Velodyne HDL 32E device. Although the intensity information is also captured, only geometrical information (3D coordinates of points) is employed for data processing. The developed algorithm automatically identifies the tunnel’s main axis, which is then utilized for the extraction of cross sections. Next, a preliminary model is generated by fitting an ellipse to each extracted cross section. The preliminary model is refined by using residual analysis and Baarda’s data snooping method to remove outliers. The final model is then generated by applying the least squares adjustment to the outlier-free data. The obtained results indicate that the tunnel’s main axis and 1551 cross sections at 0.1 m intervals are successfully extracted. Cross sections have an average semi-major axis of 7.8508 m with a standard deviation of 0.2 mm and an average semi-minor axis of 7.7509 m with a standard deviation of 0.1 mm. The modeling results show that the dimensions of cross sections are quite consistent suggesting a minimal variation in the tunnel’s cross sectional shape and dimensions. The average normal distance of all points from the constructed model (average absolute error) is also 0.012 m. Moreover, since subway tunnels are either elliptic- or circular-shaped and a circle is a specific form of an ellipse (with equal semi-major and semi-minor axes), the developed algorithm is applicable to tunnels with either elliptic or circular cross sections. The proposed methodology is also applicable to tunnels with any horizontal orientation and degree of curvature since it makes no assumptions, nor does it use any priori knowledge regarding the curvature and horizontal orientation of the tunnel.

This study can be pursued in three aspects: construction progress monitoring (in project management); displacement and deformation analysis (maintenance applications); and for the planning of future design enhancement. The construction progress can be monitored by comparing the as-built and as-designed models of a tunnel. The displacement and deformation can be analyzed by: scanning the tunnel in two or more epochs; generating the tunnel’s as-built model in each epoch using the developed algorithm; and comparing the dimensions of extracted cross sections (for deformation analysis) and 3D coordinates of cross sections center (for displacement analysis). Deformation and displacement analyses require the data from different epochs to be either in the same local reference system or in a national/global reference system. Finally, a tunnel’s as-built model can be used for the planning of future design enhancement since it provides a very precise and accurate survey of its current state.

## Figures and Tables

**Table 1:** Specifications of the Velodyne 32E LiDAR sensor.

Parameters	Value
Dimensions	0.145 m × 0.086 m
Measurement rate	700,000 (points/s)
Measurement range	100 m
Positional accuracy	0.02 m
Angular resolution	1.33°

**Table 2:** Point Sampling on Various Parts of the Tunnel.

Object	Sampling (Points per/m^2^)
Side wall	1426
Ceiling	509
Floor (around rail tracks)	652

**Table 3:** Average Dimensions of the Extracted Cross Sections.

Statistics	Mean	Standard Deviation
Semi-major axis (m)	7.8508	0.0002
Semi-minor axis (m)	7.7509	0.0001
Area (m^2^)	95.5838	0.0003
Eccentricity	0.17606	0.00002
